# Does Empirically Derived Classification of Individuals with Subjective Cognitive Complaints Predict Dementia?

**DOI:** 10.3390/brainsci9110314

**Published:** 2019-11-07

**Authors:** Eduardo Picón, Onésimo Juncos-Rabadán, Cristina Lojo-Seoane, María Campos-Magdaleno, Sabela C. Mallo, Ana Nieto-Vietes, Arturo X. Pereiro, David Facal

**Affiliations:** 1 Department of Methodology of Behavioral Sciences, University of Santiago de Compostela, 15782 Santiago de Compostela, Galicia, Spain; eduardo.picon@usc.es; 2 Department of Developmental Psychology, University of Santiago de Compostela, 15782 Santiago de Compostela, Galicia, Spain; onesimo.juncos@usc.es (O.J.-R.); cristina.lojo@usc.es (C.L.-S.); maria.campos@usc.es (M.C.-M.); sabelacarme.mallo@usc.es (S.C.M.); arturoxose.pereiro@usc.es (A.X.P.)

**Keywords:** cognitive aging, mild cognitive impairment, subjective cognitive complaints, dementia, cluster analysis, Compostela aging study, screening and diagnosis

## Abstract

(1) Background: Early identification of mild cognitive impairment (MCI) in people reporting subjective cognitive complaints (SCC) and the study of progression of cognitive decline are important issues in dementia research. This paper examines whether empirically derived procedures predict progression from MCI to dementia. (2) Methods: At baseline, 192 participants with SCC were diagnosed according to clinical criteria as cognitively unimpaired (70), single-domain amnestic MCI (65), multiple-domain amnestic MCI (33) and multiple-domain non-amnestic MCI (24). A two-stage hierarchical cluster analysis was performed for empirical classification. Categorical regression analysis was then used to assess the predictive value of the clusters obtained. Participants were re-assessed after 36 months. (3) Results: Participants were grouped into four empirically derived clusters: Cluster 1, similar to multiple-domain amnestic MCI; Cluster 2, characterized by subjective cognitive decline (SCD) but with low scores in language and working memory; Cluster 3, with specific deterioration in episodic memory, similar to single-domain amnestic MCI; and Cluster 4, with SCD but with scores above the mean in all domains. The majority of participants who progressed to dementia were included in Cluster 1. (4) Conclusions: Cluster analysis differentiated between MCI and SCD in a sample of people with SCC and empirical criteria were more closely associated with progression to dementia than standard criteria.

## 1. Introduction

Mild cognitive impairment (MCI) is a condition characterized by cognitive impairment with minimal effects on instrumental activities of daily living (IADL). Although MCI can represent the first cognitive expression of Alzheimer disease (AD), it can also be secondary to other disease processes (i.e., other neurological, neurodegenerative, systemic or psychiatric disorders) [1]. MCI is considered as a stage of the cognitive continuum, which is traditionally divided into three stages: cognitively unimpaired (CU), MCI and dementia [2]. The 2018 NIA-AA working group [2] updated the standard core criteria [3,4,5] for diagnosing MCI as a cognitive syndrome, as follows: (1) cognitive performance below the expected range based on population norms; (2) evidence of cognitive impairment that may be reported by the individual or by an observer; (3) cognitive impairment may be characterized by presentations that are not primarily amnestic; (4) neurobehavioral disturbance may be a prominent feature of the clinical presentation, e.g., changes in mood, anxiety or motivation; and (5) independent performance of daily life activities, but cognitive difficulty may result in detectable but mild functional impact on more complex activities of daily life. The definition is applicable to all individuals who meet these core criteria regardless of biomarker profile. In addition, MCI has been classified into four subtypes of cognitive impairment: single-domain amnestic MCI (sda-MCI), which requires the presence of amnestic deficits in isolation; single-domain non-amnestic (sdna-MCI), which includes single-domain non-amnestic deficits; multidomain amnestic MCI (mda-MCI), with deficits in multiple cognitive domains, including memory impairment; and multidomain non-amnestic MCI (mdna-MCI), characterized by deficits in multiple cognitive domains except memory [1,4,5].

Some authors have proposed the use of cluster analysis or latent class analysis to classify MCI and to examine how individuals are grouped together on the basis of patterns of performance of different measures, with no requirement for fulfillment of the predetermined standard criteria [6,7,8,9,10,11,12,13]. These data-driven classifications have yielded various MCI subtypes that do not always coincide in different studies, because the measures used and the criteria for using them are not always the same and the samples differ in size and characteristics.

In a cluster analysis of 134 MCI participants diagnosed with the standard criteria [4,5], Clark and colleagues [8] used a cut-off of 1.5 or more standard deviations (SDs) below normative means on at least one measure in the battery of neuropsychological tests. These researchers included 13 neuropsychological measures in the cluster analysis: four measures for verbal memory, two for executive functioning, three for language, two for visuospatial/visual memory and two for attention. They identified three distinct groups: an amnestic/language MCI subgroup based on mildly impaired memory and verbal fluency performance; a mixed or multi-domain MCI subgroup based on impaired scores on measures of memory, executive function, language and visuospatial function; and a subgroup of participants who performed within normal limits on all measures. The same researchers conducted another cluster analysis of 80 participants who were diagnosed with MCI by using the same criteria but considering low levels of performance (defined as greater than 1 SD below normative means) on at least two measures within a cognitive domain [8]. They identified four MCI subgroups: dysexecutive, individuals with impairments in measures of executive function, verbal fluency, attention, and visuospatial abilities (but with intact memory function); amnestic, individuals who were mildly impaired on delayed recall and one recognition measure, but performed within normal limits on other measures; mixed, individuals who exhibited impaired scores on measures of memory, executive function, language and visuospatial function; and the fourth subgroup, which performed approximately one standard deviation below the normative means on a single measure of visuospatial function. 

In another relevant study, Hanfer and colleagues [11] used a latent class design to analyze 1655 patients diagnosed as MCI according to standard criteria [5]. These researchers used seven cognitive measures for overall cognitive status, executive functioning, language, attention and episodic memory. They identified four cognitively different subgroups: (1) minimally impaired, a subgroup indistinguishable from the cognitively normal group; (2) amnestic only, characterized by a subtle impairment in delayed memory; (3) amnestic multi-domain, characterized by impairments across cognitive domains including memory, language and executive function; and (4) executive function and language impairments, a subgroup distinguished neuropsychologically by impairment in non-memory domains. 

In a recent study, Edmonds and colleagues [10] performed cluster analysis on data from 825 participants diagnosed with standard criteria as MCI and used six neuropsychological measures that were administered to all participants. Three different domains of cognitive ability were assessed: language, attention/executive function and memory. Four MCI subtypes were distinguished: dysnomic, with a significant deficit in naming; dysexecutive, with a significant deficit in executive function, as well as impairments in attention, naming and memory; amnestic MCI, with isolated memory impairment; and a cluster-derived normal group of participants, who performed within the normal range for attention/executive function and memory.

Four main subtypes of MCI were identified in the above-mentioned studies: multi-domain amnestic or mixed MCI, with impairment in several cognitive domains including memory; amnestic or single-domain amnestic MCI, with impairments only in memory; multi-domain non-amnestic, also called dysexecutive/language MCI, with impairments in executive functions, language, attention, visuospatial domains but not in memory; and a minimally impaired group, a subgroup almost indistinguishable from the cognitively normal group. Edmonds and colleagues concluded that the presence of one MCI subgroup almost indistinguishable from the normal group indicates the susceptibility of the conventional criteria for mild cognitive impairment to false-positive diagnostic errors [10]. However, as other researchers have also concluded the empirically derived classification of MCI based on cluster subtypes is quite consistent with the classification based on standard criteria [7].

The prognosis for patients diagnosed with MCI regarding progression to dementia is one of the most important aspects of clinical work and research [14,15] and the debate regarding how rate of progression is related to MCI diagnosis and subtypes is still open [1,2]. A recent review (a meta-analysis of high confidence, multiple concordant Class I studies) by the Subcommittee of the American Academy of Neurology [1] concluded that although patients diagnosed as MCI by using standard criteria have a higher risk of progressing to dementia than their age-matched controls, many individuals diagnosed with MCI may remain stable or return to neurologically intact. However, individuals who revert remain at a higher risk of progression back to MCI or dementia than those who have never been diagnosed with MCI. Hendfelt and colleagues [12] reported that when using empirical criteria (latent class analysis), the minimally impaired MCI class showed the slowest decline over time, and that the other three MCI classes (amnestic only, amnestic multi-domain and non-amnestic (executive-language)) were more likely to convert to probable dementia than the minimally impaired MCI subgroup. Edmonds and colleagues [10] reported that the lowest rate of progression to probable dementia occurred in the cluster-derived normal group. 

The revised studies did not compare the utility of empirically derived classification of MCI with the conventional/standard criteria to predict dementia. In order to obtain further information about the differential progression from MCI to dementia, the aim of the present study was to examine whether diagnosis of MCI subtypes by using standard and empirical criteria predicts progression to dementia in a sample of people attending primary care centers with subjective cognitive complains. We compared the utility of using empirically derived classification of MCI subtypes versus conventional/standard methods for predicting deterioration or stability over time in early stages of the cognitive continuum, such as subjective cognitive complains and MCI. 

## 2. Materials and Methods

### 2.1. Participants

The study included 205 participants aged ≥50 years who completed baseline and follow-up assessments within the Compostela Aging Study, an ongoing longitudinal project on cognitive decline carried out in public Primary Care Health Centers in Santiago de Compostela and Vigo, Spain [16]. The participants were referred to the project by general practitioners and were included on the basis of the following criteria: (1) the presence of subjective cognitive complaints (participants spontaneously reported that their cognition was not as good as before), and (2) no prior diagnosis of cognitive impairment, clinical stroke, traumatic brain injury, motor-sensory defects, alcohol or drug abuse/dependence, or diagnosis of any significant medical or psychiatric illnesses. 

All participants underwent extensive evaluation, including a medical history review and neuropsychological assessment. As an indicator of health status, the Charlson Comorbidity Index [17] was obtained from the medical history. A follow-up assessment was conducted at around 36 months after the baseline assessment (mean follow-up, 37.49 months; standard deviation, 5.34; range, 28–57 months).

MCI was diagnosed in accordance with Petersen/Winblad criteria [4,5], as revised by Albert et al [3]: (i) evidence of concern corroborated by an informant about a change in cognition, in comparison with the previous level; (ii) evidence of poorer performance in one or more cognitive domains that is greater than expected for the patient’s age and educational background; (iii) preservation of independence in functional abilities; and (iv) non-fulfillment of diagnostic NINCDS-ADRDA and DMS-IV criteria for dementia. For diagnosis, conducted during the baseline assessment, we evaluated subjective cognitive complaints, general cognitive performance and cognitive performance in several domains, such as language, attention, visuospatial, verbal and visual episodic memory, executive functioning and working memory, as well as processing speed. The assessment included various neuropsychological tests (see Table 1), some of them were used for MCI diagnosis with the Petersen/Winblad criteria because we have Spanish normative data for age and education, and others for cluster analyses, which we will explain in the statistical analysis subsection. A functional assessment was conducted using the Spanish version of the Lawton and Brody Index [18] to evaluate Instrumental Activities of Daily Living (IADL).

Following the detailed neuropsychological evaluation (Table 1), the MCI patients were classified into three groups according to Petersen/Winblad criteria: 40 were classified as multiple domain amnestic (mda-MCI), 24 as multiple domain non-amnestic (mdna-MCI) and 68 as single domain amnestic (sda-MCI). The amnestic MCI patients fulfilled the following criteria: memory complaints corroborated by an informant, as assessed by the Subjective Memory Complaints Questionnaire (SMCQ); performance of 1.5 SDs or more than 1.5 SDs below age norms in one measure, Short Delay Free Recall (SDFR) from the Spanish version of the California Verbal Learning Test (CVLT), which evaluates short and long delay recall of words; no significant impact on activities of daily living, as assessed by the Lawton and Brody Index [18]; and not demented, according to the National Institute of Neurological and Communicative Disorders and Stroke–Alzheimer’s Disease and Related Disorders Association (NINCDS-ADRDA) and Diagnostic and Statistical Manual of Mental Disorders-Fourth Edition criteria. With respect to general cognitive functioning, the mda-MCI patients scored less than 1.5 SDs below age and education-related norms in the Mini-Mental State Examination (MMSE) and on at least two cognitive subscales of the CAMCOG-R. Individuals were diagnosed with mdna-MCI when they fulfilled the same criteria as the mda-MCI, except in memory. 

A sample of 73 participants who performed within normal limits on memory, general cognitive functioning and specific cognitive domain tests was classified as cognitively normal. These participants were considered the control group for comparison with the MCI groups. 

Written informed consent was obtained from all participants prior to the study, which met with the approval of the Research Ethics Committee of the Xunta de Galicia (Spain) and was conducted in accordance with the provisions of the Declaration of Helsinki, as revised in Seoul 2008.

### 2.2. Measures

Only six measures of the neuropsychological assessment were included in the cluster analysis (see Table 1). These measures represent common instruments used to assess different cognitive domains typically impaired in early stages of AD or other forms of dementia (i.e., general cognitive performance, verbal episodic memory, visual episodic memory, language, working memory and processing speed). For the cluster analysis, we selected one measure from each cognitive domain to maintain a good ratio of cases per variable and to prevent over-fitting the data [33]. The variable for general cognitive performance was the total score obtained in the Spanish version of Mini-Mental State Examination (MMSE). Long Delay Free Recall (LDFR) scores from the Spanish version of the California Verbal Learning Test (CVLT) were used as indicators of verbal episodic memory. Pattern Recognition Memory (percentage of correct responses) (from the Cambridge Neuropsychological Test Automated Battery: CANTAB) was used to assess visual episodic memory. The Language subscale score of the Spanish version of the Cambridge Cognitive Examination (CAMCOG-R) was used as a joint variable for language (comprehension, naming, verbal fluency, definition, repetition and reading/writing). Among components of executive function, working memory (i.e., the capacity to maintain and simultaneously manipulate and control information) is usually one of the most severely impaired cognitive functions in MCI [34]. Span tasks have been used in participants with different levels of cognitive impairment, normal controls and patients with MCI or AD [35]. We used the Counting Span task and considered the longest span series completed without mistakes as the corresponding variable. Processing speed was measured by the 5-choice reaction time test in the CANTAB Automated Battery. The 5-choice reaction time (RTI) test is a latency test in which the participant must react as soon as a yellow dot appears in one of the five possible locations.

### 2.3. Statistical Analysis

The data analyzed were the *z* scores for all 6 variables that were statistically derived (SPSS Statistics 24, IBM corporation, Chicago, IL, USA) because we did not have normative data for all of them. Higher *z* scores represent better performance on all variables except RTI, in which higher RT represents greater cognitive slowing.

Ten of the 205 participants (initial sample) had missing values in some of the considered variables and were therefore excluded from the sample. The scores for the remaining 195 participants were initially analyzed by the single-linkage procedure (SPSS Statistics 24, IBM corporation) in order to identify potential outliers [36]. Three outliers were detected and excluded from the analysis. The final sample thus comprised 192 subjects. The main defining characteristics of the final sample are shown in Table 1.

We proceeded to carry out a two-stage cluster analysis [37,38] to identify profile subtypes. In the first stage, we performed a hierarchical cluster analysis, and in the second stage, we performed an iterative cluster analysis. We opted to use Euclidean distances as a measure of similarity due to the metric nature of the data [39] and Ward’s algorithm as the conglomerate method, as this is more powerful than other agglomerative clustering techniques as it uses F values to maximize differences between clusters [39,40]. We checked the coefficients (within-cluster sum of squares) in the agglomeration scheme to search for significant changes at each combination step [39]. The dendrograms provided support to the quantitative criterion for obtaining the optimal number of clusters. Both procedures produced the optimal result, and the solution was composed of four conglomerates. This result was validated by execution of a second hierarchical analysis (method of within-group average linkage), another one of the most recommended cluster methods from a statistical point-of-view. The highest percentage of correspondence between Ward and within-group linkage—in terms of the assignment of the subjects to the groups—was obtained with the solution of four groups (69%), showing the high convergent validity of the four clusters obtained.

In the second stage of the clustering process, we conducted an iterative k-means procedure (SPSS command) using the means of the four-cluster solution as starting points (seeds). We used this procedure to improve assignment of participants to clusters and to obtain the optimal solution. Iterative procedures, such as the k-means, are more powerful and reliable than hierarchical procedures, but require prior specification of the number of clusters and of the initial centers. We chose to include the means obtained through the within-group linkage due to the smaller number of reassignments to other segments that had to be performed by k-means. Only 13% of individuals in the sample were reassigned to another cluster, thus demonstrating the stability of the solution. MANOVA was conducted to further check the robustness of these clusters, revealing a significant multivariate cluster effect (Wilks’ λ = 0.081, F(18,518) = 41.312, *p* < 0.001, η^2^ = 0.568). 

The clusters obtained were compared in relation to the socio-demographic and neuropsychological variables not included in the cluster analysis. Gender was analyzed by cross tabulation and χ^2^ was used to obtain global significance and corrected standardized residuals [41] in order determine the corresponding associations. The other variables were tested by either ANOVAs and post-hoc Games–Howell tests, recommended for unequal groups in size [42], or by Kruskal–Wallis non-parametric analysis. 

In order to determine whether the cluster solution predicts conversion to dementia, we chose a categorical regression as implemented in the CATREG module of the SPSS 24 CATREG model [41], as a more reliable and preferable regression technique to the more common logit models (as it can be run confidently with fewer assumptions). We were interested in determining which predictor (groups identified using standard criteria or empirical criteria) best explained the dependent variable, rather than in determining the proportion of variance explained by the whole model. As predictors, we used two categorical variables: groups derived using standard criteria, with four values (mda-MCI, mdna-MCI, sda-MCI and HC), and groups derived using empirical criteria, with four values (Cluster 1, Cluster 2, Cluster 3 and Cluster 4). Conversion to dementia was the dependent variable with two values: converters and non-converters. 

Correspondence analysis was executed for graphical display of the contingency tables and multivariate categorical data.

## 3. Results

### 3.1. Groups Derived by Cluster Analysis

**Figure 1:**
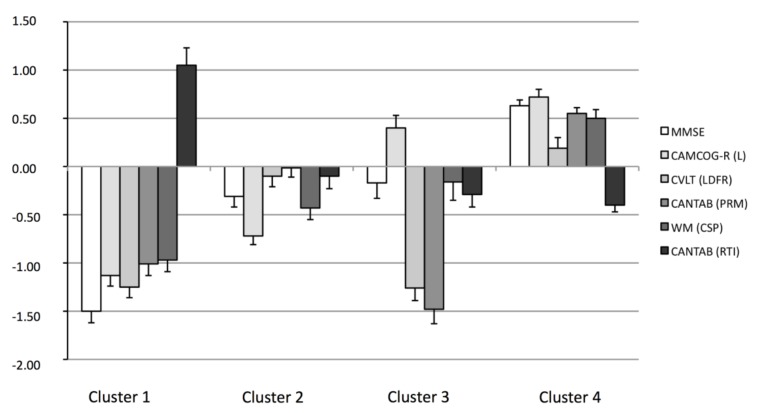
Mean values (*z* scores) and standard errors (bars) of the six neuropsychological measures used for the cluster analysis for the four empirical groups (Cluster 1, Cluster 2, Cluster 3 and Cluster 4). MMSE: Mini-Mental State Examination; CAMCOG-R (L): Cambridge Cognitive Examination (Language); CVLT (LDFR): California Verbal Learning Test (Long Delay Free Recall); CANTAB (PRM): CANTAB (Pattern Recognition Memory); WM (CSP): Working Memory (Counting Span); CANTAB (RTI): CANTAB (RTI, Five Choice Reaction Time)

We identified four optimal clusters of participants who were grouped according to their similarity in the set of the six considered variables. We performed ANOVAs and post hoc Games–Howell tests because of the different sizes of the groups [41], to test differences between clusters for each of the variables. The means and standard deviations (*z* scores), F values and size of effect (partial eta-squared) are shown in Table 2, and the level of significance is indicated. Raw scores are displayed in Supplementary Material Table S1. Graphical mean results (*z* scores) are shown in Figure 1. 

Participants grouped in Cluster 1 were characterized by the highest level of cognitive deterioration, with scores ranging from 1.50 to 0.97 SD below the mean on general cognitive performance (MMSE), language (CAMCOG-R, Language), episodic verbal memory (CVLT, Long Term Free Recall), episodic visual memory (CANTAB, Pattern Recognition Memory) and working memory (Working Memory, Counting Span), as well as the highest degree of processing slowing (0.88 SD above the mean in CANTAB, Five Choice Reaction Time). Participants in Cluster 1 were therefore classified as having amnestic multidomain MCI. Cluster 4 included participants who performed best on all domains, with scores above the mean for general cognitive performance, language, episodic verbal and visual memory and working memory, and below the mean for processing slowing. Therefore, participants in Cluster 4 were classified as cognitively unimpaired (CU). Participants in Cluster 2 scored below mean for processing slowing (0.18 SD below the mean) and for general cognitive performance (MMSE), language and working memory (respectively 0.31, 0.72 and 0.43 SDs below the mean), although with relatively good preservation of episodic verbal and visual memory, with scores close to the mean (0.10 and 0.01 SD below the mean, respectively). However, the scores did not reach the cut-off scores for MCI diagnosis. This group may represent cognitively unimpaired individuals who demonstrate weaknesses in language and working memory but not on verbal and visual episodic memory. Participants in Cluster 3 showed deterioration on verbal episodic memory (1.26 SD below the mean) and visual episodic memory (1.48 SD below the mean). By contrast, the general cognitive performance and working memory performance of these individuals were slightly below the mean values (0.17 and 0.16 SD) and their performance in language (0.40 SD above the mean) and processing slowing (0.41 SD below the mean) were well preserved. Participants in Cluster 3 were therefore classified as having amnestic single-domain MCI.

The characteristics and comparison of the four clusters on demographic and neuropsychological variables are shown in Table 3 (raw measures). Participants in Clusters 1 and 3 were older than those in Clusters 2 and 4. The level of education of participants in Clusters 1 and 2 was lower than that of the those in Clusters 3 and 4. No significant differences were found in gender distribution, comorbidity (Charlson Index) or functionality (Lawton and Brody Index). Subjective cognitive complaints were higher for Cluster 1 than for the other three clusters. General cognitive performance, evaluated by CAMCOG-R total scores, decreased progressively from Cluster 4 to Cluster 1. With respect to specific domains, Cluster 1 performed worst on all measures. Cluster 2 performed significantly worse than Cluster 4 on attention/calculation, praxis-visuospatial, executive function (CAMCOG-R), verbal fluency and vocabulary (Peabody), but scored similarly to Cluster 4 in verbal memory (CVLT, short delay free recall) and visual memory (CANTAB, PAL errors adjusted, six shapes). Cluster 3 performed significantly worse than Cluster 4, mainly on memory (CVLT, short delay free recall) and visual memory (CANTAB, PAL errors adjusted, six shapes), but also on executive function and verbal fluency. 

The four empirically classified groups show some degree of correspondence with the groups diagnosed by standard criteria. However, the correspondence is only of 52.08%, affecting only to 100 of 192 participants (Table 4, see elements of the diagonal). 

Participants diagnosed as multiple-domain amnestic MCI were reasonably well assigned to Cluster 1, with 75.75% (25/33) of correspondence; only 9.10% (3/33) and 15.15% (5/33) were assigned to Cluster 2 and Cluster 3, respectively. A large majority (91.67%) of multiple-domain non-amnestic MCI (mdna-MCI) patients were distributed between Cluster 2 (66.77%, 16/24) and Cluster 1 (25%, 6/24) and only one participant (4.16%) was allocated to each of Cluster 3 and Cluster 4. Most (88.57%) of the Healthy Control (HC) participants were distributed between Cluster 4 (61.43%, 43/70) and Cluster 2 (27.14%, 19/70), whereas 4.28% (3/70), and 7.11% (5/70) were assigned to Cluster 1 and Cluster 3, respectively. However, the single-domain amnestic MCI (sda-MCI) group was distributed almost equally among the four clusters, 20% (13/65) to Cluster 1, 24.61% (16/65) to Cluster 2, 24.61(16/65) to Cluster 3 and 30% (20/65) to Cluster 4. Column-wise analysis of the results reveals a low correspondence between Cluster 2 and the standard MCI groups, as the cluster included 16 participants with mdna-MCI, 16 with sda-MCI and 19 healthy controls.

### 3.2. Cluster Groups and Prediction of Progression to Probable Dementia

Table 4 also includes the number of participants (total = 24) classified by standard and empirical criteria at baseline who progressed to dementia 36 months after the base-line evaluation. The “total standard criteria” column shows the number of participants who progressed to dementia in each standard criteria group. The number of participants who progressed to dementia in each of the four clusters is included in the “total cluster criteria” row. Results of the cross-tabulation analysis are shown in Table 4 and the number of participants belonging to each standard or cluster group who significantly progressed to dementia is indicated. Significance was determined by statistically significant adjusted standardized residuals (after χ^2^) and is represented as +/−, which indicates that the number of people who progress to dementia is greater or lower (positive or negative value for adjusted standardized residuals) than expected by chance. Cluster 1 included most of the participants who significantly (more than randomly expected) progressed to dementia (15/24, 62.50%), while according to standard criteria these participants were mainly distributed between the mda-MCI group (10/24, 41.66%, significantly) and the sda-MCI group (10/24, 41.67%, not significantly). The number of patients who progressed from Cluster 2 (2/24, 8.33%) and from Cluster 4 (3/24, 12.50%) was significantly lower than expected due to chance. Cluster 3 included 16.66% (4/24, not significant) of participants who progressed to dementia.

Cross-tabulation analysis was used to test the association between the standard criteria and the empirical criteria with progression to dementia, revealing that both criteria were significantly associated with progression to dementia. However, the values of the Chi-square statistic (χ^2^ (3, *n* = 192) = 23.72, *p* < 0.001) and the Cramer’s V statistic (V = 0.35, *p* < 0.001) for the empirical criteria were higher than the corresponding values (χ^2^ (3, *n* = 192) = 17.90, *p* < 0.001; V = 0.30, *p* < 0.001)) for the standard criteria. 

The categorical regression analysis (CATREG), that included the two variables (the standard and empirical criteria groups) as predictors and progression to dementia as dependent variables, showed that both variables significantly predicted progression to dementia (Table 5). However, the values of β, F, *r* (partial correlation with progression to dementia) and P (Pratt’s relative importance) for the groups classified by empirical criteria were higher than those for the groups classified by standard criteria, indicating that the former was a better predictor than the latter. 

**Figure 2:**
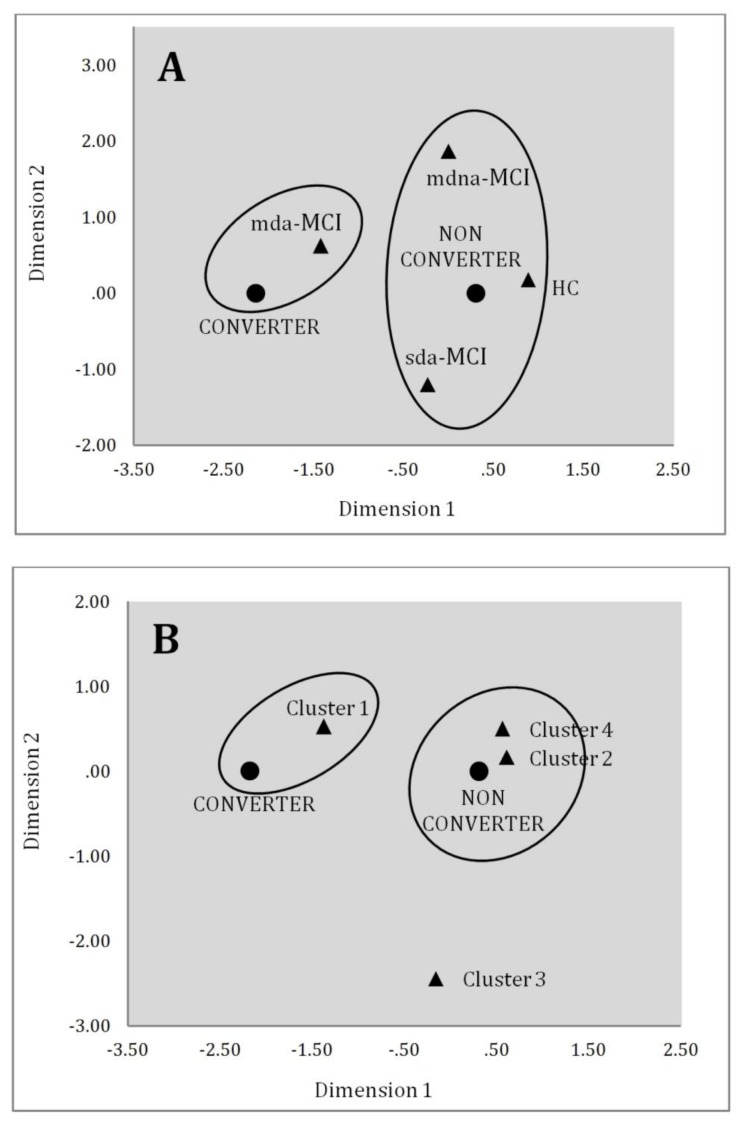
Scatter plot of category points in the multiple correspondence analysis for the associations between evolution of dementia and general impairment in the clinically diagnosed groups (**A**) and the clustering solution (**B**).

Correspondence analysis was performed to produce a graphical display of the contingency tables and multivariate categorical data. The standard mda-MCI group and the empirically derived Cluster 1 were clearly associated with the “converter to dementia” value (Figure 2). However, the association with the “non-converter” value is not as clear. While the other three standard groups (mdna-MCI, sda-MCI and particularly HC) were associated with the non-converter group, only the empirically derived Clusters 2 and 4 appeared closer to the non-converter group. Cluster 3 appeared significantly further away from all others cluster and from both converter and non-converter groups.

## 4. Discussion

In this study we empirically classified a sample of participants with subjective cognitive complaints into subgroups by applying cluster analysis to participants’ scores on six neuropsychological measures and compared the empirical and standard criteria on progression to dementia. Four clusters were identified. Socio-demographical comparisons did not reveal any differences between clusters in gender, health status (comorbidity) or functionally; however, the oldest participants were included in Clusters 1 and 3. According to their cognitive profiles, Cluster 1 and Cluster 3 appear to correspond respectively to the standard multiple-domain and single domain amnestic MCI subgroups. Cluster 4 included participants with no cognitive impairments, although all of these participants presented subjective cognitive complaints and their scores in the corresponding questionnaire were not different from those obtained by participants groups in Clusters 2 and 3. Therefore, the individuals in Cluster 4 appear to have subjective cognitive decline (SCD) as they fulfilled the three main criteria for diagnosis of SCD recommended by the Subjective Cognitive Decline Initiative (SCD-I) [43]: (1) self-reported persistent decline in cognitive capacity in comparison with a previously normal status, and not related to an acute event; (2) normal performance on standardized cognitive tests, which are used to classify mild cognitive impairment (MCI); and (3) decline cannot be explained by a psychiatric or neurologic disease (apart from AD), medical disorder, medication, or substance use.

The profile of participants included in Cluster 2 deserves special consideration: These were the youngest participants (together with those from Cluster 4) and they performed below the mean in language and working memory, but they did not reach the cut-off score for diagnosis of multiple-domain non amnestic MCI or language/dysexecutive MCI. We suggest that Cluster 2 includes individuals who, although they do not meet the MCI criteria, are undergoing early cognitive decline, especially in language and executive function. These participants may be considered as having SCD with incipient objective cognitive decline in several domains except episodic memory. 

These results are partly consistent with those of other empirical studies showing that the two main amnestic MCI subgroups (single and multiple domains) can be classified by cluster analysis [7,8,10,11]. Although we did not clearly identify a dysexecutive/language subgroup, interestingly, the participants in Cluster 2 performed poorly on many non-memory measures but did not reach MCI cut-off scores. However, the definition of a dysexecutive/language group varies widely and different empirical studies have reported some heterogeneity in neuropsychological profiles. Clark and colleagues [8] identified an amnestic/language MCI subgroup, based on mildly impaired memory and verbal fluency performance, and another dysexecutive (non-amnestic) subgroup with impairments in executive function, verbal fluency, attention and visuospatial abilities (but with intact memory function). Edmonds and colleagues [10] identified a dysnomic subgroup with significant deficits in naming and a dysexecutive (amnestic) subgroup with significant deficits in executive function, as well as impairments in attention, naming and memory. In turn, Hanfer and colleagues [11] described a dysexecutive/language subgroup with impairments in executive function and language. 

The particular characteristics of our study, involving a sample of people attending primary care centers with subjective cognitive complaints, may have contributed to the formation of two clusters, Cluster 4 and Cluster 2, which could be defined as subgroups with subjective cognitive decline (SCD). Cluster 4 could be a proper SCD group and Cluster 2 a SCD group with incipient non amnestic MCI, representing two different levels on the cognitive continuum from normal aging to dementia that may be useful for categorization of SCD as prior manifestation to the onset of clinical impairment [44,45]. However, further research is required to produce a more precise definition of these two clusters. Although these participants fulfilled the three core criteria for SCD [43], their scores were not compared with those of an appropriate control group (cognitively unimpaired and no subjective cognitive complaints reported), which was not available in the present study due to sampling constraints. In addition, other more precise psychometric determinations of the measures of subjective cognitive complaints as well as other evaluations of complex functionality, mood and personality [46] must also be taken into account. Our research team has recently studied early affective and behavioral impairments included in a syndrome called mild behavioral impairment [47], which includes symptoms such as decreased drive/motivation, affective/emotional dysregulation, impulsive dyscontrol and agitation, social inappropriateness and delusions and hallucinations, observing a low prevalence of these impairments in individuals with SCD [48].

Similarities between the groups classified using empirical and standard criteria indicated that Cluster 1 included 75.76% of individuals diagnosed as multiple-domain amnestic MCI. This group captured individuals with more subjective cognitive complaints and with the highest level of impairments in several cognitive domains, i.e., older patients with more advanced MCI. The correspondence between this MCI subgroup identified using empirical and standard criteria has also been reported in previous studies [6,7,8,9,10,11,12,13]. Individuals diagnosed using standard criteria as having multiple-domain non-amnestic MCI corresponded mainly to Cluster 2 (66.67%), but also to Cluster 1 (25%). The correspondence between Cluster 2 and multiple-domain non-amnestic MCI is consistent with our interpretation of this cluster as a subgroup of participants with incipient impairment in several cognitive domains, mainly language, attention and executive functions, but not memory. We suggest that the other participants (25%) allocated to Cluster 1 may correspond to individuals with amnestic and non-amnestic impairments, who are difficult to classify with standard criteria due to the difficulty involved in applying cut-off scores in some neuropsychological measures. Edmonds and colleagues [10] also identified a dysexecutive (amnestic) subgroup with a significant deficit in executive function, as well as impairments in attention, naming and memory. 

Participants included in Cluster 3 presented a cognitive profile with specific deficit in verbal and visual episodic memory consistent with the standard subtype of single-domain amnestic MCI. However, their correspondence with this subtype only reached 24.62%. The remaining 75.38% of patients with single-domain amnestic MCI was distributed almost equally among Clusters 1, 2 and 4. This low level of correspondence was also observed in other empirical studies: for example, Clark and colleagues [8] found that only 26.47% (9/34) of participants diagnosed as single-domain amnestic MCI (standard comprehensive criteria) converged to their amnestic subgroup (cluster criteria); and Edmonds and colleagues [10] found that only one-third of their MCI patients (standard criteria) were solely amnestic, with another third representing primarily dysnomic or dysexecutive/multiple-domain amnestic subtypes. We suggest that many patients classified as single-domain amnestic with standard criteria may have mild deficits in other cognitive domains that are not captured with these criteria, and others may have memory deficits that are not critical enough to reach the MCI stage. A subgroup (around 25%) of participants diagnosed with single-domain amnestic MCI could be considered as having SCD and could revert to unimpaired cognitive ability, as observed in some studies [49]. We also suggest that differences between groups categorized using standard and empirical criteria in different studies may be due to the type and number of variables included in the analyses, the type and size of the samples, and the ratio of cases per variable, in order to prevent the risk of over-fitting the data. We believe that a meta-analysis study should be undertaken to examine all of these aspects and to draw conclusions about the correspondence. 

The rates of progression to probable dementia shown in Table 1 are consistent with cognitive characterization of the clusters. Cluster 1, which encompassed individuals at a more advanced stage of MCI included most of the participants who significantly (more than randomly expected) progressed to dementia. The rate of progression in Cluster 3 (only amnestic MCI) was low and not significant; the rates were also low and significantly lower than expected for respectively Cluster 2 (initial stage of multiple-domain non amnestic MCI) and Cluster 4 (subjective cognitive decline). From the point-of-view of progression, these groups are consistent with the concept of deterioration as a cognitive continuum usually divided into different stages, such as unimpaired cognition, subjective cognitive decline, mild cognitive impairment and dementia [1,2,46]. According to standard criteria, the two amnestic MCI groups (multiple and single domain) included 83.33% of the participants who progressed to dementia, whereas the multiple-domain non- amnestic MCI only included 12.5% of such participants. Despite the differences in the rates of progression between the groups classified using standard and empirical criteria, our results are consistent with the most recent guidelines indicating amnestic MCI subtypes as the most likely to progress to dementia [1]. The results of the cross-tabulation and categorical regression analyses showed that both criteria were significantly associated and predicted progression to dementia; however, empirical criteria appeared to be more closely associated and are also better predictors than standard criteria. Indeed, graphical analysis of the matches shows that both the multiple-domain amnestic MCI group and Cluster 1 are associated with the subsequent progression of the individual members to dementia. The differences arise in the interpretation of the other groups. Thus, although in the standard classification the other groups (single-domain amnestic MCI, multiple-domain non-amnestic MCI and controls) did not tend to progress to dementia, in the empirical classification only Clusters 4 and 2 appear clearly linked to no progression (and much closer than for the standard criteria). On the other hand, Cluster 3 appears significantly further away from all other groups and from the predictors, showing that the members of this group were not linked, neither to progression nor to no progression. This result may be interpreted as a confirmation that Cluster 3 and Cluster 1 represent two points of severity along the continuum from mild cognitive impairment to dementia as in the corresponding standard MCI groups, single- and multiple-domain amnestic MCI subtypes [50]. 

Finally, several study limitations should be noted. The current study is based on a comprehensive battery of neuropsychological tests. However, it does not include information on biomarkers in blood and cerebrospinal fluid or on structural and functional neuroimaging markers, which would probably enhance our understanding of the differences underlying the clusters and their progression to dementia. Future studies within the ongoing longitudinal project will include biological and neuroimaging markers to complete and validate the current findings. Additionally, the current study did not include a group correctly characterized as cognitively unimpaired and without subjective cognitive complains. We are aware that it is difficult to find adults over 50 years old without any subjective cognitive complaints, but in the near future the characterization of subjective cognitive complaints as a cognitive stage different from cognitively unimpaired decline should be refined taking into account the recommendations of the Subjective Cognitive Decline Initiative (SCD-I) Working Group [43,46]. Despite these limitations, we believe that the current findings add some insight into the stages of the cognitive continuum from normal aging to dementia and in their progressive decline, particularly in the stage called Subjective Cognitive Decline. We also think that the comparative findings on the groups classified by standard and empirical criteria will provide clinicians and researchers with a better understanding of the complementarity of both methods for defining the different stages of cognitive profiles and predict progression to dementia. Clinicians, who use the standard criteria more frequently, could correct their diagnoses using empirical criteria. They could use standard criteria for a first diagnosis of individual patients and then perform clustering analysis with other neuropsychological measures, stored as data not used in the clinical diagnosis, to determine their allocation to the corresponding clusters. Researchers should analyze through meta-analysis of empirical studies, which are the measures that best define the different stages of the cognitive continuum, and then use them as predictors of progression to dementia in longitudinal studies that include also biological and neuroimaging markers, as well as affective and behavioral symptoms. 

## 5. Conclusions

Using empirically derived methods, four subgroups have been identified. Individuals included in Cluster 1 performed between 1 and 1.5 SD below the mean level on all measures. Those in Cluster 2 scored below the mean level (less than 1 SD lower), particularly in general cognitive performance, language and working memory. Participants included in Custer 3 scored around 1.5 SD below the mean level only in episodic memory measures. Finally, those participants included in Cluster 4 performed above the mean on all measures. According to their cognitive profile, Cluster 2 deserves special consideration as a SCD group with incipient non-amnestic MCI. Participants included in Cluster 3 presented specific deficits in verbal and visual, but their consistence with the single-domain amnestic MCI subtype was low. Cluster 1, which encompassed individuals at a more advanced stage of MCI, included most of the participants progressed to dementia. Although cross-tabulation and categorical regression analyses showed that both empirical and clinical criteria significantly predicted progression to dementia, empirical criteria appeared to be more closely associated and are also better predictors of these progressions.

## Figures and Tables

**Table 1:** Demographic and neuropsychological characteristics (raw measures, means and standard deviations in parentheses) of the participants (*n* = 205) diagnosed using clinical standard MCI criteria: Group 1 = multiple-domain amnestic MCI (mda-MCI), Group 2 = multiple-domain non amnestic MCI (mdm-MCI), Group 3 = single-domain amnestic MCI (sda-MCI), and Group 4 = healthy controls (HC). Statistical measures (ANOVA F, Kruskal–Wallis non-parametric H and standard χ^2^), effect size (ŋ^2^ and Cramer V), and post-hoc comparisons of groups 1–4). The variables shown in italics were included in the cluster analysis.

	mda-MCI (Group 1) *n* = 33	mdna-MCI (Group 2) *n* = 24	sda-MCI (Group 3) *n* = 65	HC (Group 4) *n* = 70	Test ^a^	Effect Size ^b^	Distinct Groups ^c^
Demographic variables							
Age	70.88 (7.94)	65.95 (8.94)	68.83 (9.25)	67.13 (8.92)	*F* = 1.96	0.030	
Gender (*n* = F/M)	25/8	21/3	31/34	46/24	χ^2^ = 15.34 **	0.283	2 +female 3 +male
Years of Education	9.67 (3.97)	7.00 (3.24)	9.54 (4.21)	10.37 (5.25)	*H* = 6.76	0.035	
Comorbidity (Charlson Index)	1.21 (1.24)	1.50 (1.31)	0.85 (0.91)	0.83 (0.93)	*H* = 6.93	0.036	
Tests for MCI diagnosis							
Lawton and Brody	6.68 (1.81)	6.00 (1.62)	6.95 (1.68)	7.14 (1.22)	*F* = 2.16	0.053	
SMCQ (Informant)	17.40 (4.67)	18.48 (4.98)	16.75 (4.49)	13.46 (3.91)	*F* = 9.35 **	0.158	1, 2, 3 > 4
SMCQ (Patient)	19.36 (4.53)	20.96 (3.62)	19.11 (4.75)	13.09 (1.76)	*H* = 96.25 **	0.504	1, 2, 3 > 4
MMSE	22.97 (1.91)	24.04 (2.05)	27.12 (1.77)	28.36 (1.34)	*H* = 107.66 **	0.564	1, 2 < 3 < 4
CAMCOG-R (Total)	70.92 (10.47)	73.75 (6.15)	82.57 (9.01)	87.45 (9.02)	*H* = 67.62 **	0.352	1, 2 < 3 < 4
CAMCOG-R (Orientation)	8.21 (1.39)	9.21 (0.72)	9.37 (0.74)	9.73 (0.48)	*H* = 45.20 **	0.237	1 < 2, 3 < 4
CAMCOG-R (Attention/Calculation)	5.12 (2.19)	4.25 (1.82)	7.35 (1.63)	7.59 (1.50)	*F* = 34.81 **	0.357	1, 2 < 3, 4
CAMCOG-R (Praxis-visuospatial)	9.55 (2.22)	9.58 (1.72)	10.80 (1.36)	11.04 (1.15)	*H* = 23.89 **	0.125	1, 2 < 3, 4
CAMCOG-R (Executive function)	13.22 (3.93)	13.83 (3.34)	15.92 (4.28)	18.52 (4.71)	*F* = 16.03 **	0.193	1, 2 < 3 < 4
Verbal Fluency (Animals)	11.35 (3.73)	13.12 (3.36)	14.77 (4.77)	18.05 (5.63)	*H* = 46.00 **	0.239	1 < 2, 3 < 4
Peabody (Vocabulary)	52.47 (17.88)	45.62 (12.98)	58.25 (17.71)	64.73 (17.94)	*F* = 8.72 **	0.121	1, 2 < 3, 4
CVLT (Short Delay Free Recall)	3.27 (2.14)	8.96 (2.14)	4.00 (2.01)	10.04 (3.09)	*H* = 126.67 **	0.663	1, 3 < 2, 4
Tests for cluster analysis							
CAMCOG-R (Language)	22.94 (2.25)	22.92 (1.91)	24.91 (2.40)	25.67 (2.66)	*F* = 13.93 **	0.182	1, 2 < 3, 4
CVLT (Long Delay Free Recall)	4.12 (3.30)	9.42 (2.87)	5.35 (2.92)	10.74 (3.12)	*F* = 53.35 **	0.460	1, 3 < 2, 4
CANTAB (Pattern Recognition Memory)	67.17 (10.46)	78.12 (11.21)	76.79 (12.28)	83.33 (12.23)	*F* = 14.12 **	0.184	1 < 3, 2, 4
Counting Span (Working Memory)	1.67 (1.14)	2.00 (1.10)	2.08 (1.30)	2.61 (1.22)	*F* = 5.21 **	0.077	1 < 4
CANTAB (RTI, Five-choice Reaction Time)	500.15 (126.99)	407.19 (64.16)	427.75 (100.60)	399.14 (94.65)	*H* = 20.09 **	0.105	1> 2, 3,4

** *p* < 0.01. SMCQ = Subjective Memory Complaints Questionnaire [19]; MMSE = Mini-Mental State Examination [20,21]; CAMCOG-R = Cambridge Cognitive Examination [22,23,24,25]; Peabody [26,27]; CVLT = California Verbal Learning Test [28,29]; CANTAB = Cambridge Neuropsychological Test Automated Battery [30]; Counting Span [31,32]. ^a^, *F* (3188), *H* (3, *n* = 192) and χ*^2^*(3, *n* = 192). ^b^, ŋ*^2^*with *F* and *H, V* with χ*^2^*. ^c^, Tukey HSD test (after F test), Mann–Whitney U (H) or adjusted standardized residuals (χ^2^).

**Table 2:** Neuropsychological characteristics (*z* scores) of variables used for cluster analysis (means and standard deviations in parentheses) and comparisons of the four empirical groups (Cluster 1, Cluster 2, Cluster 3 and Cluster 4).

Variables	Cluster 1 *n* = 47	Cluster 2 *n* = 54	Cluster 3 *n* = 27	Cluster 4 *n* = 64	Test ^a^	Effect Size ^b^	Distinct Groups ^c^
MMSE	−1.50 (0.86)	−0.31 (0.79)	−0.17 (0.84)	0.63 (0.50)	H = 106.58 **	0.558	1 < 2, 3 < 4
CAMCOG−R(Language)	−1.13 (0.80)	−0.72 (0.64)	0.40 (0.66)	0.72 (0.67)	F = 82.40 **	0.568	1 < 2 < 3, 4
CVLT (Long Delay Free Recall)	−1.26 (0.80)	−0.10 (0.80)	−1.26 (0.67)	0.19 (0.86)	F = 41.61 **	0.399	1, 3 < 2, 4
CANTAB (Pattern Recognition Memory)	−1.01 (0.85)	−0.01 (0.75)	−1.48 (0.76)	0.55 (0.52)	H = 104.78 **	0.549	1, 3 < 2 < 4
Working Memory (Counting Span)	−0.97 (0.87)	−0.43 (0.90)	−0.16 (0.99)	0.50 (0.73	H = 68.09 **	0.356	1 < 2, 3 < 4
CANTAB (RTI, Five-choice Reaction Time)	0.88 (1.07)	−0.18 (0.70)	−0.41 (0.62)	−0.24 (0.64)	H = 46.91 **	0.245	1 > 2, 3, 4

**, *p* < 0.01. Note: ^a^ F Degrees of freedom (3188) and H (3, *n* = 192); ^b^ ŋ^2 c^ Post-hoc comparisons, Tukey HSD test (after F) and Mann–Whitney U (after H).

**Table 3:** Demographic and neuropsychological characteristics (raw measures, means and standard deviations in parentheses) of the participants (*n* = 192) using empirical MCI criteria for the variables not included in the cluster analysis. Statistical measures include ANOVA F, Kruskal–Wallis non-parametric H and standard χ^2^, effect size (ŋ^2^ and Cramer V) and post-hoc comparisons of Clusters 1–4.

	Cluster 1 *n* = 47	Cluster 2 *n* = 54	Cluster 3 *n* = 27	Cluster 4 *n* = 64	Test ^a^	Effect Size ^b^	Distinct Groups ^c^
Age	73.60 (7.12)	66.41 (8.26)	70.52 (9.87)	64.78 (8.34)	F = 11.87 **	0.159	1, 3 > 2, 4
Gender (*n* = F/M)	31/16	36/18	14/13	42/22	χ^2^ = 2.05		
Years of Education	8.17 (3.86)	7.46 (2.64)	10.63 (4.58)	11.85 (5.17)	H = 31.01 **	0.162	1, 2 < 3, 4
Comorbidity (Charlson Index)	0.97 (1.05)	1.01 (1.05)	1.22 (1.33)	0.85 (0.92)	F = 0.77		
Lawton & Brody	6.89 (1.69)	6.74 (1.50)	7.05 (1.61)	6.85 (1.50)	F = 0.16		
Subjective Cognitive Complaints (Informant)	17.92 (4.68)	17.02 (4.67)	17.05 (4.58)	13.38 (3.81)	F = 10.04 **	0.168	1, 2, 3 > 4
Subjective Cognitive Complaints (Patient)	18.46 (4.54)	18.46 (5.88)	17.89 (4.22)	14.87 (3.47)	H = 24.70 **	0.129	1, 2, 3 > 4
CAMCOG-R (Total)	71.34 (8.24)	78.35 (5.95)	83.59 (6.73)	91.72 (6.35)	F = 87.50 **	0.583	1 < 2 < 3 < 4
CAMCOG-R (Orientation)	8.57 (1.21)	9.31 (.75)	9.33 (1.00)	9.75 (.50)	H = 42.57 **	0.223	1 < 2, 3, 4
CAMCOG-R (Attention/Calculation)	5.28 (2.14)	6.04 (2.19)	7.26 (1.72)	7.97 (1.20)	H = 50.05 **	0.262	1, 2 < 3, 4
CAMCOG-R (Praxis-visuospatial)	9.15 (2.03)	10.48 (1.27)	10.81 (1.18)	11.43 (.94)	H = 51.92 **	0.272	1 < 2 < 4; 1 < 3
CAMCOG-R (Executive function)	12.36 (3.44)	14.67 (3.26)	16.70 (3.95)	20.30 (3.64)	F = 50.66 **	0.447	1 < 2 < 3 < 4
Verbal Fluency (Animals)	10.47 (2.93)	13.91 (3.26)	15.56 (5.31)	19.83 (4.74)	H = 92.04 **	0.482	1 < 2, 3 < 4
Peabody (Vocabulary)	46.07 (17.60)	50.30 (13.31)	62.44 (15.24)	70.54 (14.74)	H = 62.98 **	0.330	1, 2 < 3, 4
CVLT (Short Delay Free Recall)	3.62 (2,47)	7.65 (3.10)	4.22 (2.75)	9.20 (3.76)	H = 71.93 **	0.377	1, 3 < 2, 4
CANTAB(PAL errors adjusted-6 shapes)	29.26 (15.40)	13.49 (8.71)	21.96 (15.73)	9.62 (7.53)	H = 51.19 **	0.268	1 > 3 > 2, 4

** *p* < 0.01. CAMCOG-R = Cambridge Cognitive Examination; CVLT = California Verbal Learning Test; CANTAB = Cambridge Neuropsychological Test Automated Battery; PAL = Pair associated Learning. ^a^ F (3188), H (3, *n* = 192) and χ^2^ (3, *n* = 192). ^b^ ŋ^2^ with F and H, V with χ^2^. ^c^ Tukey HSD test (after F test), Mann–Whitney U (H).

**Table 4:** Cross-tabulated data for standard criteria groups and empirical criteria groups (clusters). Number of participants corresponding to standard criteria groups correctly allocated to the empirical groups (Allocat.). Number of participants that progressed to probable dementia in each group (Progres.).

			Empirical Criteria Groups (Clusters)				Total Standard Criteria	% Correct ^a^
			1	2	3	4		
Standard criteria groups	Multiple-domain amnestic MCI (mda-MCI)	Allocat	25	3	5	0	33	75.76
		Progres.	9^+^	0	1	0	10^+^	90.00
	Multiple-domain non-amnestic MCI (mdna-MCI)	Allocat.	6	16	1	1	24	66.67
		Progres.	1	2^+^	0	0	3	66.67
	Single-domain amnestic MCI (sda-MCI)	Allocat.	13	16	16	20	65	24.62
		Progres.	5	0	3	2	10	30.00
	Healthy control (HC)	Allocat.	3	19	5	43	70	61.43
		Progres.	0	0	0	1^+^	1^−^	100.00
Total cluster criteria		Allocat.	47	54	27	64	192	
		Progres.	15 ^+^	2^−^	4	3^−^	24	
% Correct ^a^			53.19	29.63	59.26	67.19		52.08
			60.00	100.00	75.00	33.33		62.50

^a^ Values can be considered indicators of convergent validity (correspondence between groups): % of the total sample correctly assigned to the diagonal (i.e., 75.76 = 25/33 × 100) and % of participants showing progression to dementia assigned to the diagonal (i.e., 90.00 = 9/10 × 100). ^+^/^−^ shows (positive or negative) statistically significant adjusted standardized residuals (after χ^2^). In other words, 15^+^ indicates that this group of 15 participants with dementia is significantly more than expected due to chance.

**Table 5:** Results of the categorical regression analysis (CATREG). Values for predicting conversion to probable dementia of the classification based on standard criteria and classification based on empirical criteria (cluster).

Predictor	β	F_(3,185)_	*r* (Partial Correlation)	*p* (Pratt´s Relative Importance)
Standard criteria groups	0.17	6.75 **	0.16	0.34
Empirical criteria groups (clusters)	0.27	9.46 **	0.25	0.66

** *p* < 0.01.

## Supplementary Materials

The following are available online at https://www.mdpi.com/2076-3425/9/11/314/s1, Table S1: Neuropsychological characteristics (raw scores) of variables used for cluster analysis, means and standard deviations in parentheses) and comparisons of the four empirical groups (Cluster 1, Cluster 2, Cluster 3 and Cluster 4).
