# Supplementary material for: Does Empirically Derived Classification of Individuals with Subjective Cognitive Complaints Predict Dementia?
Source: Brain Sci. 2019 Nov 7;9(11):314. doi: 10.3390/brainsci9110314 (PMC6895967; doi:10.3390/brainsci9110314)
Supplement: Supplementary file 1 [file brainsci-09-00314-s001.pdf]

Table S1. Neuropsychological characteristics (raw scores) of variables used for cluster analysis, means and standard deviations in parentheses) and comparisons of the four empirical groups (Cluster 1, Cluster 2, Cluster 3 and Cluster 4).

Variables	Cluster 1 *n* = 47	Cluster 2 *n* = 54	Cluster 3 *n* = 27	Cluster 4 *n* = 64	Test [a]	Effect Size [b]	Distinct Groups [c]
MMSE	23.44(2.12)	26.37(1.94)	26.70(2.07)	28.68(1.23)	H=106.58**	.558	1 < 2, 3 < 4
CAMCOG-R(Language)	22.19(2.02)	23.24(1.62)	26.07(1.68)	26.89(1.69)	F=82.40**	.568	1 < 2 < 3, 4
CVLT (Long Delay Free Recall)	4.38(3.20)	9.91(3.20)	4.37(2.70)	10.17(3.43)	F=41.61**	.399	1, 3 < 2, 4
CANTAB (Pattern Recognition Memory)	68.52(10.63)	81.01(9.42)	62.65(9.49)	87.95(6.47)	H=104.78**	.549	1, 3 < 2 < 4
Working Memory (Counting Span)	1.25(1.07)	1.92(1.11)	2.25(1.22)	3.07(0.89)	H=68.09**	.356	1 < 2, 3 < 4
CANTAB(RTI, Five-choice Reaction Time)	526.23(123.18)	411.66(92.30)	393.04(67.47)	382.16(58.07)	H=46.91**	.245	1 > 2, 3, 4

** $p < 0.01$; Note: [a] F Degrees of freedom (3,188) and H (3, N = 192); [b] $\eta 2$. [c] Post-hoc comparisons, Tukey HSD test (after F) and Mann-Whitney U (after H).
